# Coronary-Subclavian Steal Syndrome: Percutaneous Approach

**DOI:** 10.1155/2013/757423

**Published:** 2013-07-29

**Authors:** Carina Machado, Luís Raposo, Sílvio Leal, Pedro Araújo Gonçalves, Henrique Mesquita Gabriel, Rui Campante Teles, Manuel Sousa Almeida, Miguel Mendes

**Affiliations:** ^1^Serviço de Cardiologia, Hospital do Divino Espírito Santo de Ponta Delgada, Portugal; ^2^Serviço de Cardiologia, Hospital de Santa Cruz, Centro Hospitalar de Lisboa Ocidental, Portugal

## Abstract

Coronary subclavian steal syndrome is a rare ischemic cause in patients after myocardial revascularization surgery. Subclavian artery stenosis or compression proximal to the internal mammary artery graft is the underlying cause. The authors present a clinical case of a patient with previous history of non-ST elevation myocardial infarction, triple coronary bypass, and effort angina since the surgery, with a positive ischemic test. Coronary angiography revealed a significant stenosis of the left subclavian artery, proximal to the internal mammary graft.

## 1. Introduction

The coronary subclavian steal syndrome (CSSS) was first described in 1974 and is caused by retrograde or insufficient blood flow through the internal mammary artery graft, with subsequent myocardial ischemia. Proximal atherosclerotic stenosis of the ipsilateral subclavian artery is the most frequent cause [1]. Although most cases of angina after coronary bypass graft surgery (CABG) are due to native-vessel or graft atherosclerotic disease progression, this syndrome should not be disregarded [2]. The traditional approach for this problem is surgical revascularization of the subclavian artery with a bypass graft, but percutaneous transluminal subclavian artery angioplasty has emerged as an effective alternative to surgery and it is now a widely accepted method of treating symptomatic subclavian steal syndrome. 

## 2. Case Report

The authors present the case report of a 69-year-old male patient with several cardiovascular risk factors (hypertension, hyperlipidaemia, and previous smoking) and history of coronary artery bypass grafting (CABG) 6 years before when the left internal mammary artery (LIMA) was grafted to the left anterior descending artery (LAD), saphenous vein conduit was grafted to posterior descendent artery and left radial artery grafted to the intermediary branch. No medical imaging of the aortic arch and its branches was performed before cardiac surgery.

He had recurrence of angina following the surgery (CCS class II) mainly when exerting the upper limbs. His therapy was adjusted and remained only mildly symptomatic until 2011 when he was referred to for coronary angiography because of gradually worsening exertional angina with no response to medical therapy. There were no neurological or claudication complaints. Physical examination only showed an II/VI systolic murmur. Basal EKG and blood analysis were unremarkable. On the transthoracic echocardiogram, there was only mild aortic sclerosis and good global and segmental systolic left ventricle function. 

He had a positive exercise electrocardiographic stress test, showing a 1.5 mm ST segment depression on anterior and inferior leads accompanied by chest pain on 2nd stage Bruce treadmill protocol. 

**Figure 1:**
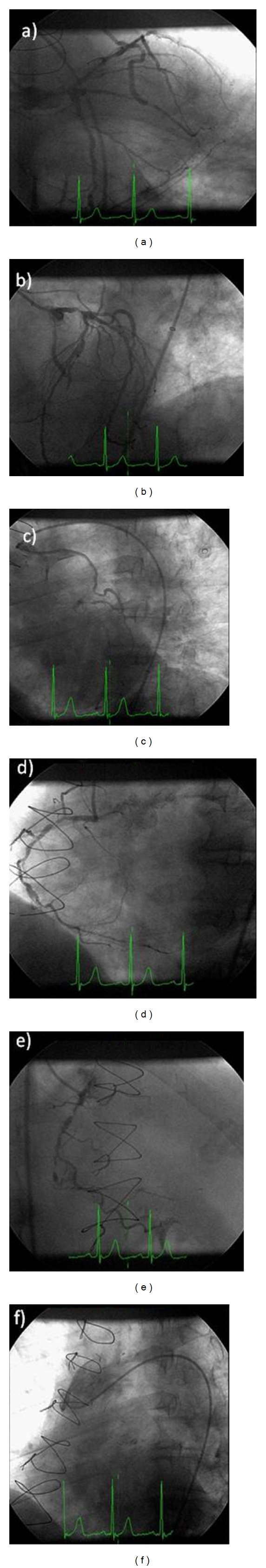
Coronary angiography showed (a) and (b) native left coronary artery territory similar to previous described and flow reversal in the LIMA graft; (c) patent left radial artery grafted in a intermediary branch; (d) and (e) native right artery territory similar to previous described; (f) saphenous vein graft to posterior descendent artery occluded.

The coronary angiography showed no new lesions in native-vessel circulation or the bypass grafts, except for the occlusion of the saphenous venous conduit grafted to the posterior descendent artery and partial retrograde filling through the LIMA graft from the LAD (Figure 1). 

**Figure 2:**
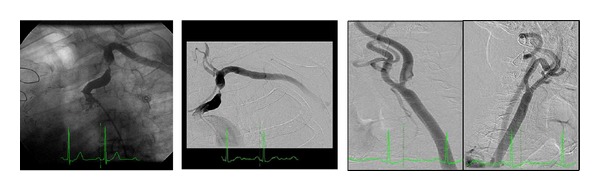
Selective left subclavian angiography showed (a) and (b) severe proximal stenosis, with slow distal flow; (c) subtraction angiography showed no lesions in the carotid arteries.

Subclavian angiography showed a proximal severe stenosis (83%: quantitative coronary angiography) with slow distal flow; there were no significant stenosis in the carotid arteries (Figure 2).

**Figure 3:**
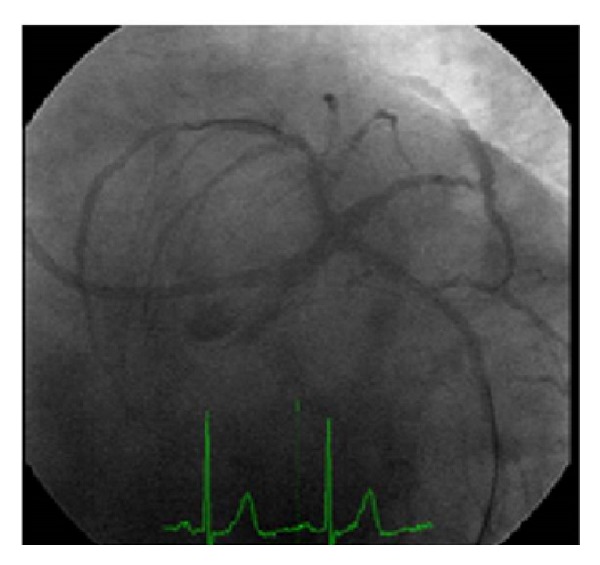
Successful ad hoc left main angioplasty, using a drug-eluting stent (Promus Element 4,0 × 12 m).

**Figure 4:**
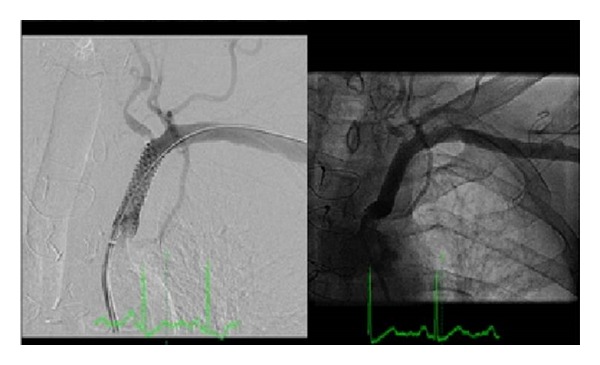
Proximal left subclavian artery angioplasty with a balloon-expanded stent (Invatec Scuba 9.0 × 30 mm—10 atm); maintained vertebral artery flow.

It was performed an ad hoc, left main percutaneous angioplasty, using a drug-eluting stent 4,0/12 mm (Promus Element) without trifurcation involvement, with final angiographic success (Figure 3). In a second procedure it was performed a proximal left subclavian artery angioplasty with a balloon-expanded stent (Invatec Scuba 9.0 × 30 mm—10 atm) (Figure 4). The procedure was uneventful. After one year of clinical followup the patient remained asymptomatic.

## 3. Discussion

Nowadays LIMA is the most used graft conduit due to excellent long-term patency, and although CSSS has a low described incidence (0.5–6.8%), perhaps this incidence could be underestimated. Ischemic symptoms can present immediately following CABG surgery or up to 7-8 years later [3, 4]. Some patients may be asymptomatic; therefore, a high level of clinical suspicion is needed, and a detailed clinical history and thorough physical exam should be performed, including measure of arterial blood pressure in both arms as a screening tool. However, several prospective studies showed no consistent positive predictive value of systolic differencial pressure in the upper arm (10–20 mmHg) [5]. Alternative screening methods can be used, such as Doppler ultrasonography, but the direct angiography remains the gold standard for the diagnosis and can be done when performing a coronary angiogram, with minimal risk. 

Controversy remains that to whether or not a routine subclavian artery angiography should be performed before CABG surgery [5]. Some authors defend that although selective subclavian artery catheterization has a low rate of complications, it still implies an additional risk for stroke or upper arm embolization [6, 7]. Nonetheless failure to identify significant subclavian disease, before placement of ipsilateral LIMA graft, may lead to the development of CSSS and myocardial ischemia [8].

The most appropriate management of concomitant brachiocephalic coronary artery disease also remains a matter of debate, but both endovascular stenting and extrathoracic surgical bypass are safe and effective treatments for CSSS in the short and medium term; extrathoracic surgical bypasses are more durable in the long term [9]. Some authors defend that endovascular stenting is less invasive, has a lower complications rate, and may result in shorter hospitalization; therefore, the preferred therapy should be considered [10]. 

## 4. Conclusion

Our case demonstrates the need for evaluating the supra-aortic branches in patients previously submitted to CABG surgery and persistent/recurrent angina symptoms, particularly in those without other causes of ischemia. The percutaneous treatment for subclavian stenosis is effective and safe.
